# Supplementary material for: Apoplastic class III peroxidases PRX62 and PRX69 promote Arabidopsis root hair growth at low temperature
Source: Nat Commun. 2022 Mar 14;13:1310. doi: 10.1038/s41467-022-28833-4 (PMC8921275; doi:10.1038/s41467-022-28833-4)
Supplement: Supplementary file 1 — Supplementary Information [file 41467_2022_28833_MOESM1_ESM.pdf]

Supplementary Information for

Apoplastic class III peroxidases PRX62 and PRX69 promote Arabidopsis root hair growth at low temperature

Javier Martinez Pacheco et al.

This File includes:

Supplementary Tables 1-3

Supplementary Figures 1-14

Supplementary Table 1. *PRX62* and *PRX69* are transcriptionally induced under low temperature.

gene name	Log2 (WT 24h/WT 0h)	Gene FDR	Tissue expression
PRX05	4,68	4,7E-21	endodermis-vascular
PRX04	3,31	1,5E-43	endodermis-vascular
PRX37	2,10	3,2E-15	endodermis-vascular
PRX62	2,04	6,2E-22	**RH**
PRX14	1,86	1,1E-05	endodermis-vascular
PRX69	1,76	1,4E-35	**RH**

FDR= False Discovery Rate. Extracted from [30] where seedlings were grown at 22°C for 9 days and then transferred to 10°C for 24 hs. RH = root hair.

Supplementary Table 2. Mutants and transgenic lines generated and used in this study.

Transgenic line name	Gene construct/ mutant lines code	References
prx62-1	GK_287E07	34
prx62-2	SALK_151762	34
prx69-1	SAIL_691_G12	34
prx69-2	SALK_137991	34
prx62-1prx69-1	*prx62-1/* GK_287E07<br>*prx69-1/* SAIL_691_G12	This study
**35Spro*PRX62*-tagRFP/Col-0 #3**	35S promoter::PRX62-tagRFP	This study
**35Spro*PRX62*-tagRFP/Col-0 #4**	35S promoter::PRX62-tagRFP	This study
**35Spro*PRX69*-tagRFP/Col-0 #1**	35S promoter::PRX69-tagRFP	This study
**35Spro*PRX69*-tagRFP/Col-0 #5**	35S promoter::PRX69-tagRFP	This study
**35Spro*PRX62*-TagRFP #1/ *prx62-1prx69-1* #4**	35S promoter::PRX62-tagRFP<br>/ *prx62-1prx69-1*	This study
**35Spro*PRX69*-TagRFP #1/ *prx62-1prx69-1* #7**	35S promoter::PRX69-tagRFP<br>/ *prx62-1prx69-1*	This study
**35Spro*PRX69*-TagRFP/ *prx62-1prx69-1 #2***	35S promoter::PRX69-tagRFP #1<br>/ *prx62-1prx69-1*	This study
**35Spro*PRX69*-TagRFP/ *prx62-1prx69-1 #6***	35S promoter::PRX69-tagRFP #1<br>/ *prx62-1prx69-1*	This study
***PRX62*proGFP**	PRX69 promoter::GFP	This study
***PRX69*proGFP**	PRX62 promoter::GFP	This study
**35SproSS-TOMATO/Col-0**	35S promoter::SS-tdTOMATO<br>/Col-0	13
**35SproSS-TOMATO-EXT-LONG/Col-0**	35S promoter::SS-tdTOMATO-EXTENSIN<br>/Col-0	13
**35SproSS-TOMATO/*prx62-1 prx69-1***	35S promoter::SS-tdTOMATO<br>/*prx62-1prx69-1*	This study
**35SproSS-TOMATO-EXT-LONG/*prx62-1 prx69-1***	35S promoter::SS-tdTOMATO- EXTENSIN<br>/*prx62-1 prx69-1*	This study
rsl4	*rsl4-1*	14
**RSL4proGFP-RSL4**	RSL4 promoter::GFP-RSL4	2
rsl2rsl4	*rsl2-1*/ SAIL_ 514C04<br>*rsl4-1*	14
**35SproRSL4**	35S promoter::RSL4	14

Supplementary Table 3. List of Primers used in this study.

Gene or Line	Primers Sequence (5'->3')	References
**genotyping by PCR**		
prx62-1	F= GATTACACACTATTAATTAGGAATTAGTTTG<br>R= GAGAGAAACCGAATCACGAG	34
prx62-2	F= GAGGAGGACACACGATC<br>R= AACGAAATTGAACTTTATTTATTCC	34
prx69-1	F= ATGGGTCGTGGTTACAATTTG<br>R= CTTGACGTCACCTTCCTTAGG	34
prx69-2	F= ATGGGTCGTGGTTACAATTTG<br>R= CTTGACGTCACCTTCCTTAGG	34
**RT-qPCR**		
PRX62	F= TCGGACCACTGTGGCATCTCA<br>R= GAGTTAGGTCCCGATAAAAGCAC	This study
PRX69	F= CTGCTGGCTGCGGTCTAGTAA<br>R= ACTTCCCTCGTCTAACTCCACT	This study
ACTIN 2	F= GGTAACATTGTGCTCAGTGGTGG<br>R= CTCGGCCTTGGAGATCCACATC	90
**35SproPRXs-tagRFP**	***PRX62***<br>F= CCCAAGCTTATGGGCTTGGTCCGAT<br>R= CGCGGATCCGCATTAACCGCAGAGC<br>***PRX69***<br>F=CCGGAATTCATGGGTCGTGGTTACA<br>R=CCGCCCGGGAGTTGATGGCGGAACA	This study

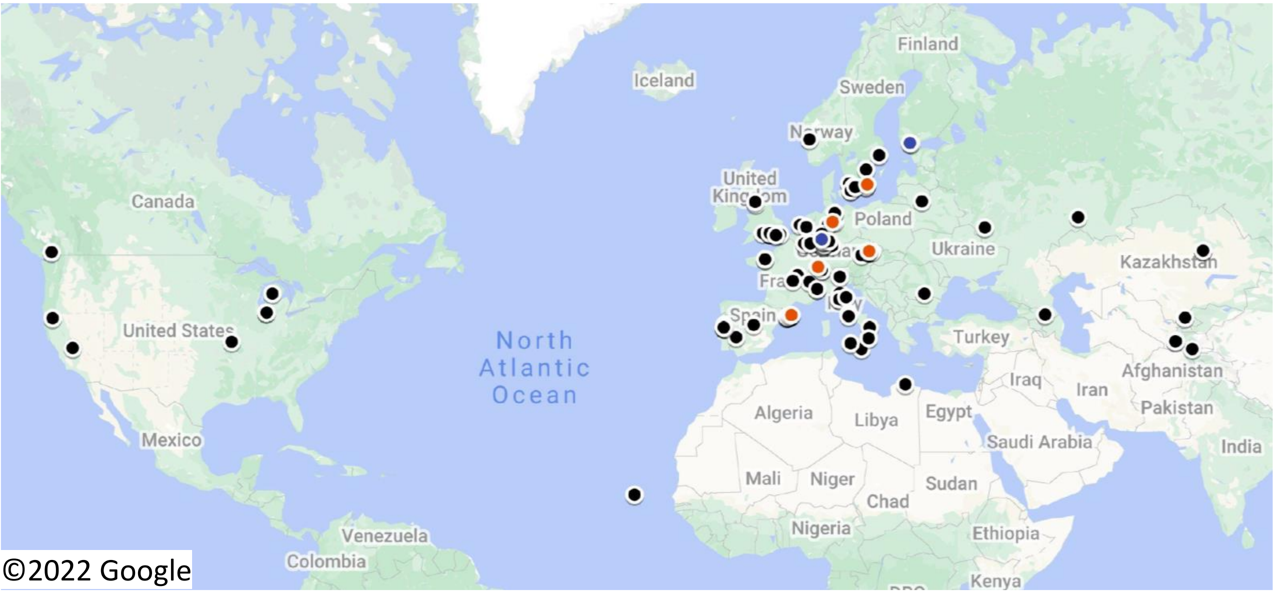

Supplementary Figure 1. Geographic location of the 108 *Arabidopsis thaliana* accessions used in this study.

Each dot represents the sampling original site of individual accession used for this study. In red color, 5 accessions with the longest RHs at 10°C, and in blue those with the shortest RHs at 10°C. Map where Arabidopsis accessions used in this study and indicated was modified from https://www.google.com/maps/d/edit?mid=1AMWwbHCx4HATjfV8UgzmvcYl_PJ22jTv&usp=sharing. Map data ©2022 Google.

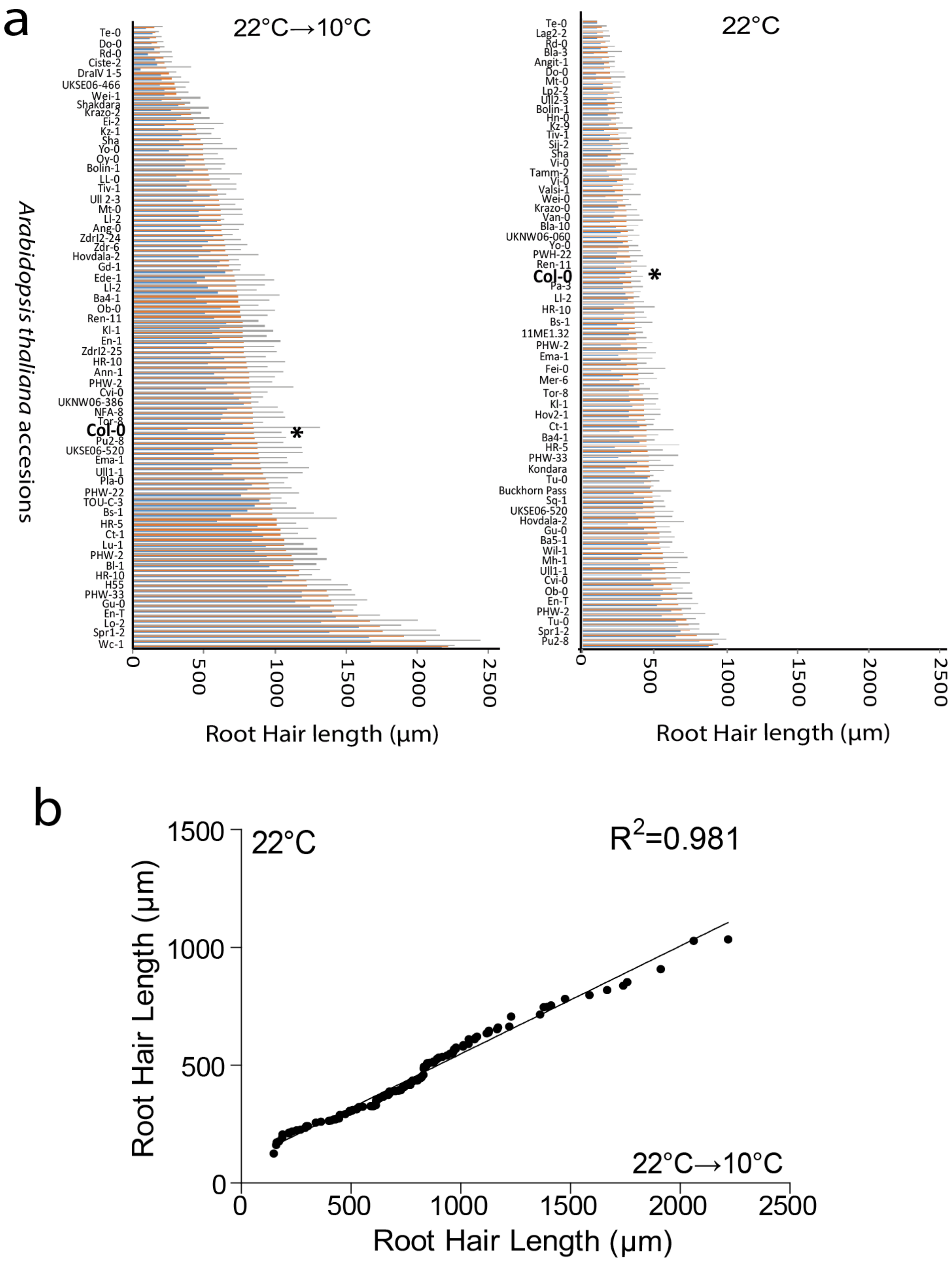
a
22°C→10°C
22°C
Arabidopsis thaliana accesions
Te-0
Do-0
Rd-0
Ciste-2
DralV 1-5
UKSE06-466
Wei-1
Shakdara
Krazo-2
Ei-2
Kz-1
Sha
Yo-0
Oy-0
Bolin-1
LL-0
Tiv-1
Ull 2-3
Mt-0
Ll-2
Ang-0
Zdrl2-24
Zdr-6
Hovdala-2
Gd-1
Ede-1
Ll-2
Ba4-1
Ob-0
Ren-11
Kl-1
En-1
Zdrl2-25
HR-10
Ann-1
PHW-2
Cvi-0
UKNW06-386
NFA-8
Tor-8
Col-0
Pu2-8
UKSE06-520
Ema-1
Ull1-1
Pla-0
PHW-22
TOU-C-3
Bs-1
HR-5
Ct-1
Lu-1
PHW-2
Bl-1
HR-10
H55
PHW-33
Gu-0
En-T
Lo-2
Spr1-2
Wc-1
*
Te-0
Lag2-2
Rd-0
Bla-3
Angit-1
Do-0
Mt-0
Lp2-2
Ull2-3
Bolin-1
Hn-0
Kz-9
Tiv-1
Sij-2
Sha
Vi-0
Tamm-2
Vi-0
Valsi-1
Wei-0
Krazo-0
Van-0
Bla-10
UKNW06-060
Yo-0
PWH-22
Ren-11
Col-0
Pa-3
Ll-2
HR-10
Bs-1
11ME1.32
PHW-2
Ema-1
Fei-0
Mer-6
Tor-8
Kl-1
Hov2-1
Ct-1
Ba4-1
HR-5
PHW-33
Kondara
Tu-0
Buckhorn Pass
Sq-1
UKSE06-520
Hovdala-2
Gu-0
Ba5-1
Wil-1
Mh-1
Ull1-1
Cvi-0
Ob-0
En-T
PHW-2
Tu-0
Spr1-2
Pu2-8
*
0
500
1000
1500
2000
2500
Root Hair length (µm)
Root Hair length (µm)
b
22°C
R2=0.981
22°C→10°C
1500
1000
500
0
0
500
1000
1500
2000
2500
Root Hair Length (µm)
Root Hair Length (µm)

Supplementary Figure 2. RH cell growth phenotype in *Arabidopsis* accessions at 22°C→10°C versus at 22°C.

(**a**) RH length phenotype in the *Arabidopsis* accessions at 22°C→10°C versus at 22°C. Average cell length on 50-300 fully elongated RHs is indicated (± SD) see Source Data file. Col-0 is indicated with an asterisk (*). Only 61 accessions are indicated in the edited graphs to improve readability. Average cell length (in orange), highest (in grey) and lowest (blue) values are shown.

(**b**) Pearson positive correlation (R^2=0.981) and linear regression fitting between RH growth of *Arabidopsis* accessions grown at 22°C versus the same accessions grown at 22°C→10°C.

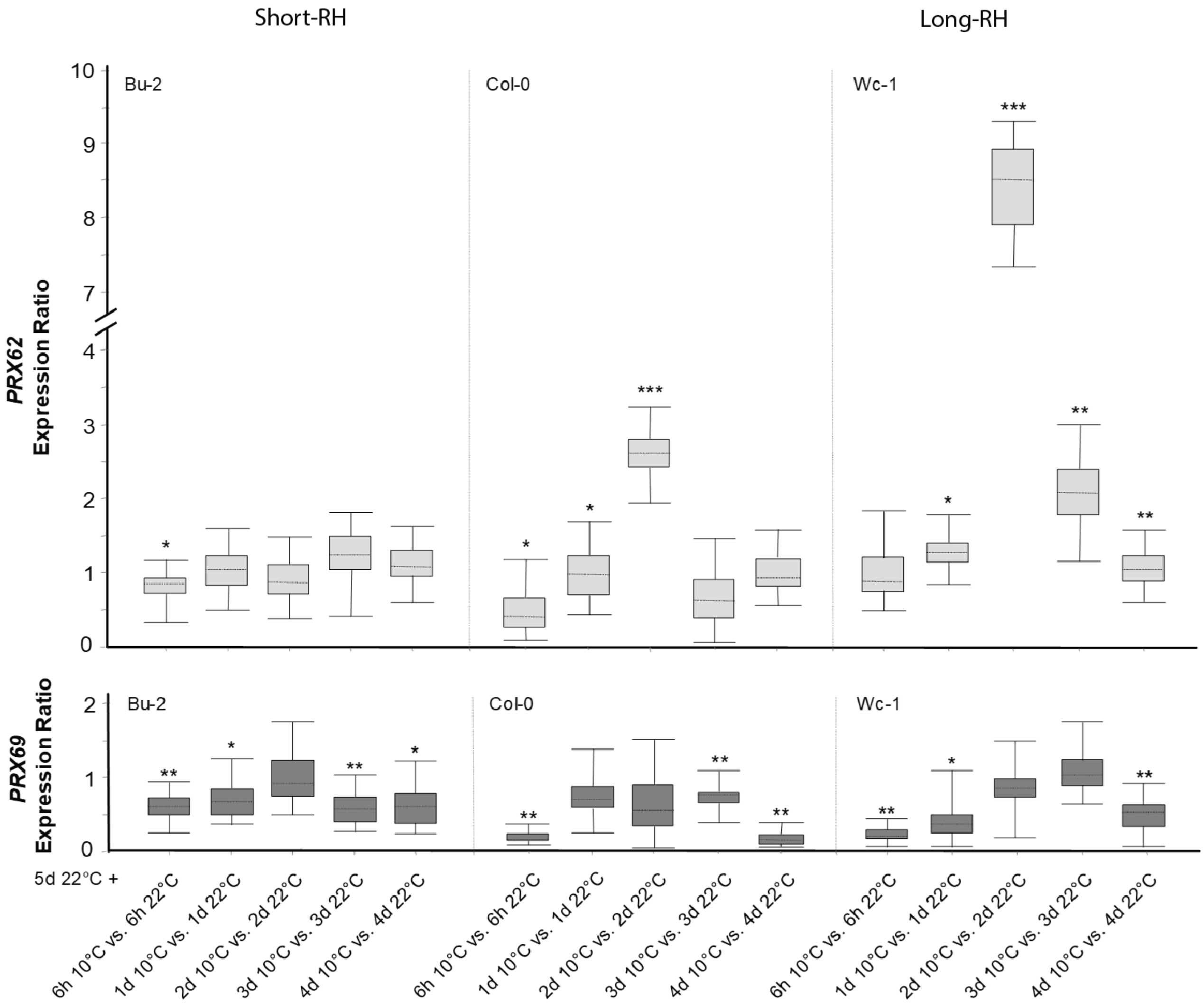

Supplementary Figure 3. ***PRX62,*** **but not** ***PRX69,*** **is differentially expressed at low-temperature (10°C) in Arabidopsis accessions with contrasting RH length phenotypes**.

Expression measured by qPCR of *PRX62* and *PRX69* in three contrasting Arabidopsis accessions based on the RH phenotype detected at 10°C. Total RNA was extracted from roots of *in vitro* plantlets (grown for 5 days at 22°C plus 6h, 1 day, 2 days, 3 days or 4 days either at 22°C or 10°C). *PRX62* and *PRX69* transcript levels determined by RT-qPCR were normalized to *ACT2* and *UBQ1* as internal controls. Boxes represent the interquartile range. The dotted line symbolizes the median gene expression. Whiskers correspond to the minimum and maximum observations (N=6). Asterisks indicate statistically significant differences between cold-treated and non-cold-treated groups (***) $p<0.001$, (**) $p<0.01$, (*) $p<0.05$. Exact *p-values* are provided in the Source Data file.

a

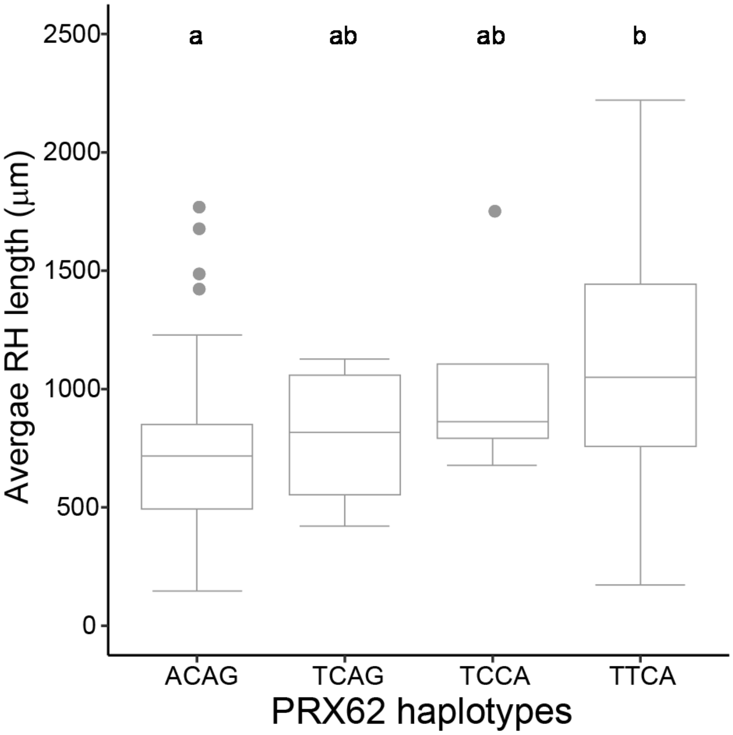

b

ANOVA analysis

Variable	N	R^2	Adj R^2	CV
Average RH length (mm)	104	0.17	0.14	48.15

S.V.	SS	df	MS	F	*p-value*
Model	2886677.81	3	962226	6.76	0.0003
Haplotype	2886677.81	3	962226	6.76	0.0003
Error	14229581.69	100	142296		
Total	17116259.5	103			

Test:Tukey Alpha = 0.05 LSD = 402.99024
Error: 142295.8169 df: 100

PRX62 haplotype	Means	n	S.E.		
TTCA	1141.96	16	94.31	a	
TCCA	1035.2	4	188.61	a	b
TCAG	795.55	4	188.61	a	b
ACAG	698.59	80	42.17		b

Supplementary Figure 4. Haplotype analysis on *PRX62* SNPs.

(**a**) Average RH length at 10°C was calculated for each informative haplotype obtained with the four SNPs identified by GWAS and localized in *PRX62*.1 coding region (number of accessions carrying each haplotype ≥ 4, total N=104).

(**b**) Model details and contrasts using one-way ANOVA. Haplotype differences were identified in a post-hoc Tukey HSD test ($p \leq 0.05$). Significant differences are indicated by different letters.

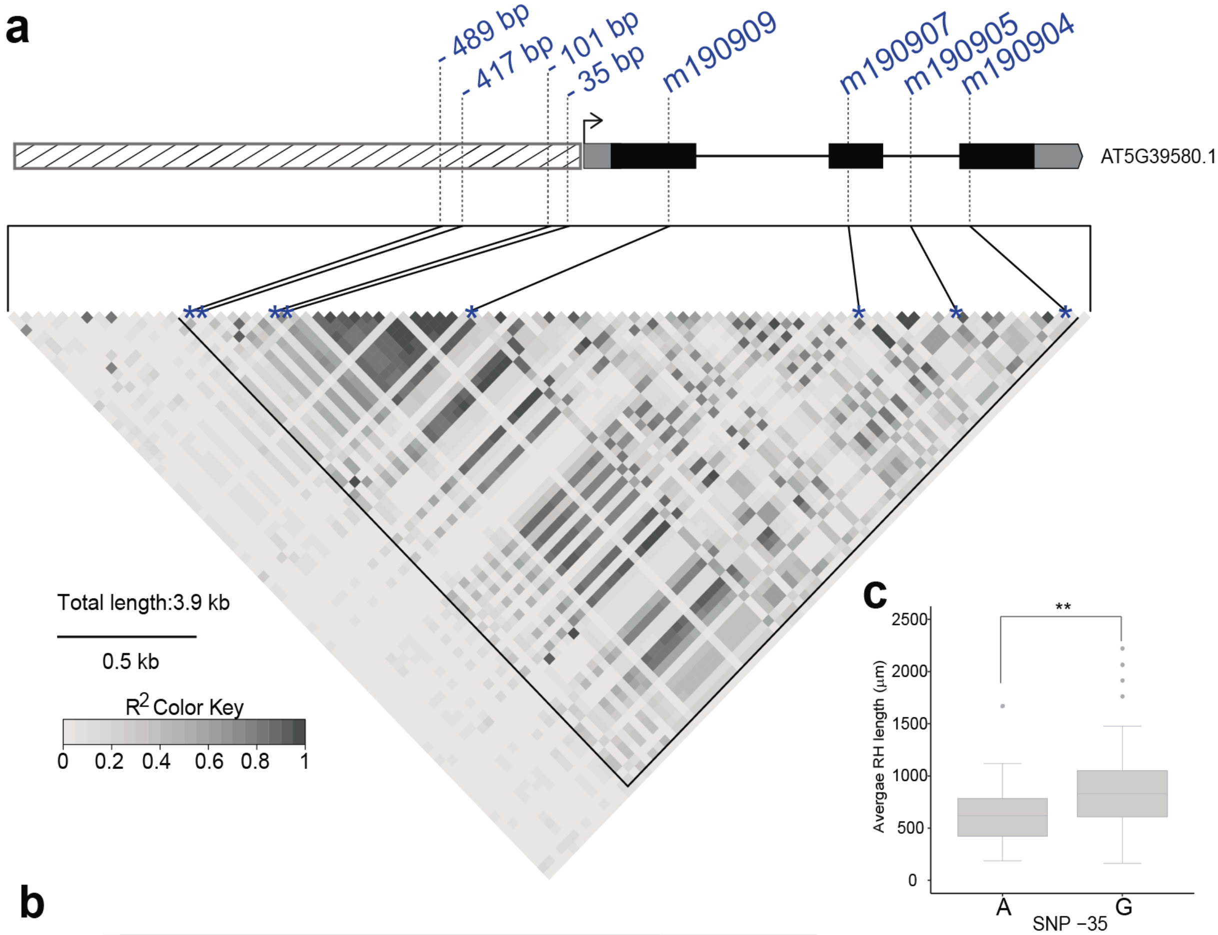

b

	m190904	m190905	m190907	m190909	SNP_-35	SNP_-101	SNP_-417	SNP_-489
m190904		0.76	0.55	0.58	0.14	0.18	0.12	0.20
m190905	0		0.64	0.61	0.11	0.14	0.09	0.25
m190907	0	0		0.49	0.08	0.11	0.07	5.49E-06
m190909	0	0	0		0.17	0.22	0.14	0.15
SNP_-35	0	0	0	0		0.71	0.19	0.03
SNP_-101	0	0	0	0	0		0.25	0.04
SNP_-417	0	0	0	0	0	0		0.02
SNP_-489	0	0	0.94	0	2.05E-08	1.92E-10	3.38E-07	

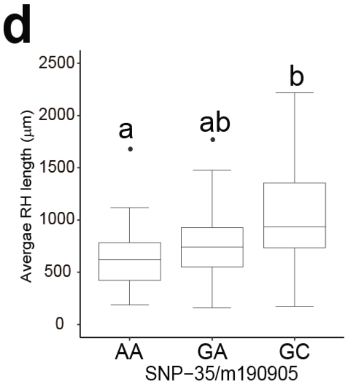

Supplementary Figure 5. Linkage disequilibrium (LD) analysis for *PRX62* locus.

(**a**) Heatmap of LD (coefficient of correlation = r^2) for SNPs in *PRX62* coding and promoter region using re-sequenced data from 1001 Arabidopsis genome (N of accessions used = 852). Gene structure, position of high-LOD SNPs detected in GWAS, and SNP in the promoter region in LD with them are shown. Distance scale (in bp) and LD scale are included in the figure.

(**b**) Table of LD values (upper diagonal) between SNPs in the promoter region of *PRX62* and the high-LOD SNPs detected in GWAS. SNPs in promoter are called according to the distance to the ATG. Lower diagonal shows the p-values for LD correlation.

(**c**) Average RH length in μm for accessions carrying different alleles at SNP-35 (n_{total}=60). *T*-test was performed to identify significant differences (** p<0.01).

(**d**) Average RH length for the haplotypes formed combining SNP-35 and the leader SNP in GWAS m190905 (N_{total}=60). Haplotype differences were identified in a post-hoc Tukey HSD test ($p \leq 0.05$) after one-way ANOVA. Significant differences are indicated by different letters.

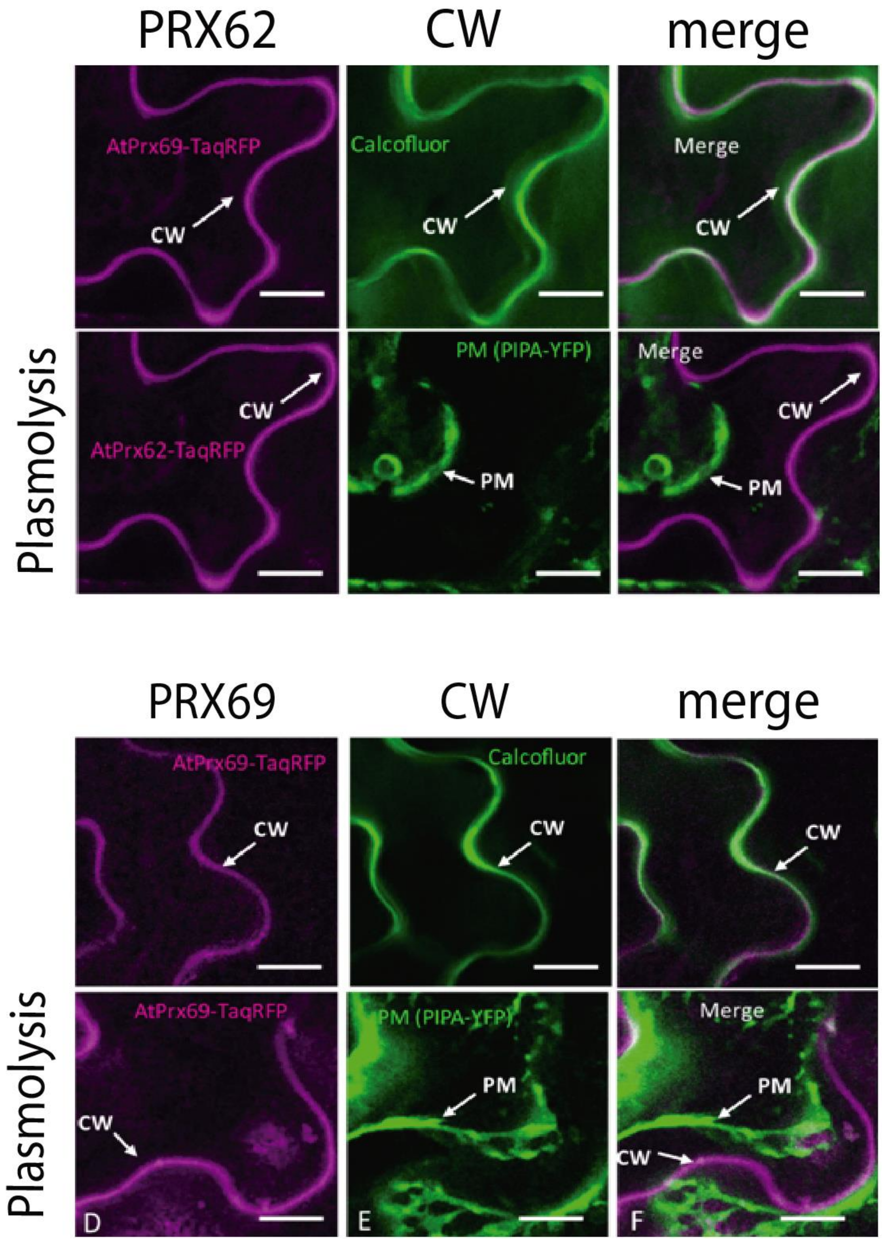

Supplementary Figure 6. **Apoplastic localization of PRX62 and PRX69.**

Confocal laser scanning fluorescence signals from *Nicotiana benthamiana* plasmolyzed leaf epidermal cells co-expressing $35S_{pro}$PRX62-TagRFP or $35S_{pro}$PRX69-TagRFP (magenta channel, left panels) together with the plasma membrane marker Yellow fluorescent protein (YFP)-tagged Plasma Membrane Aquaporin (PIP2A-YFP) (green channel, central panels). Both channels merged (white signal, right panels). The top line corresponds to a single confocal section whereas the bottom line corresponds to the maximum intensity z projection of six confocal sections. Scale bars= 50 µm. CW= cell wall; PM = plasma membrane.

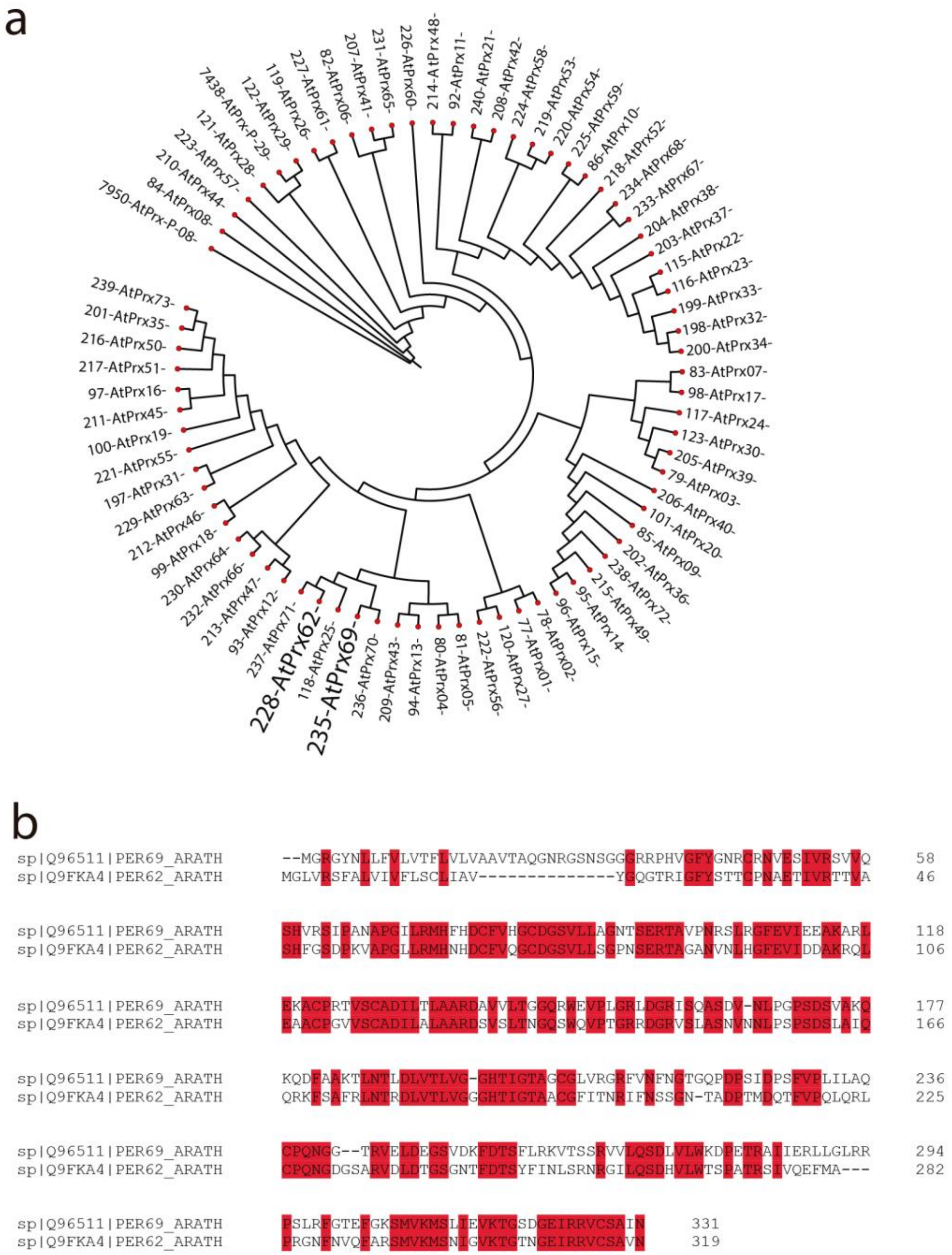

Supplementary Figure 7. **Phylogenetic tree of the 73 apoplastic class III PRXs in *Arabidopsis***.
(**a**) PRX62 and PRX69 clustered in the same subclade of the phylogenetic tree.
(**b**) High identity percentage between the PRX62 and PRX69 aminoacid sequences (57.19%). Canonical PRXs protein sequences were retrieved from UniProt Beta database (https://beta.uniprot.org/) and multiple sequence alignment was performed with Clustal O (v 1.2.4) (https://www.ebi.ac.uk/Tools/msa/clustalo/).

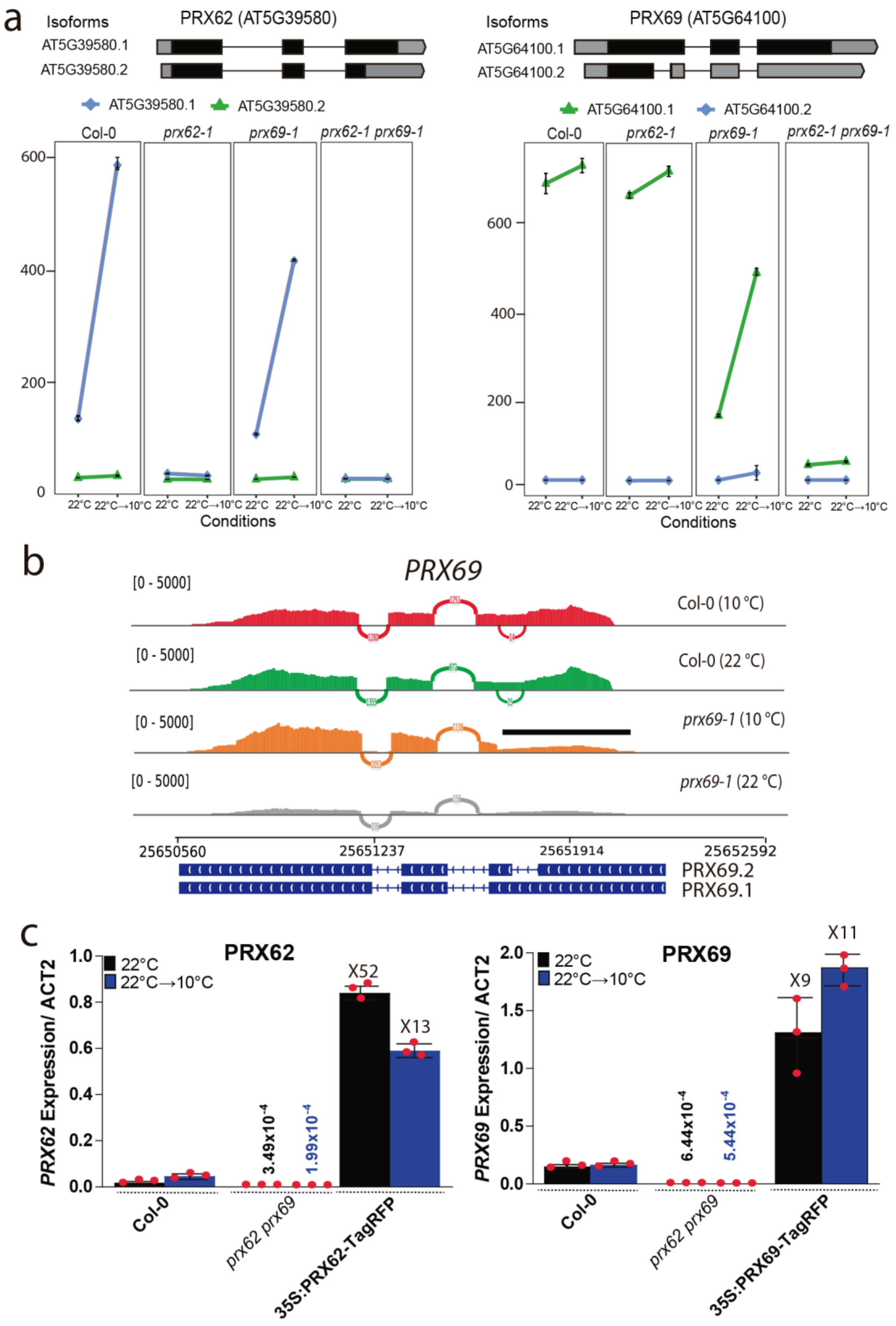

Supplementary Figure 8. Expression analysis by RNA-seq and q-PCR of *PRX62* and *PRX69*.

(**a**) Isoforms expression of *PRX62* and *PRX69* in Col-0, *prx62-1*, *prx69-1* and *prx62-1 prx69-1* double mutant determined by RNA-seq. Reads in *PRX69* gene in *prx69-1* mutant backgrounds showed a truncated version being expressed. Isoforms' schemes were adapted from boxify (https://boxify.boku.ac.at/). TPM = Transcripts Per Kilobase Million.
(**b**) Sashimi plots of *PRX69* indicate the expression of a truncated RNA in the *prx69-1* mutant. Sashimi plots (adapted from IGV) show the coverage for each alignment track (Col-0 and the *prx69-1* mutant) plotted as a histogram; arcs represent splice junctions connecting exons. Alternative splicing isoforms for *PRX69* are displayed below. The line on top of the graph highlights the region of the RNA that shows low coverage or low expression.
(**c**) Levels of *PRX62* and *PRX69* expression in Col-0 roots, *prx62-1 prx69-1* double mutant and over-expressor *PRX62* and *PRX69* lines. *ACT2* was used for normalization of gene expression. Three biological replicates and three technical replicates per experiment were performed. Exact *p-values* are provided in the Source Data file.

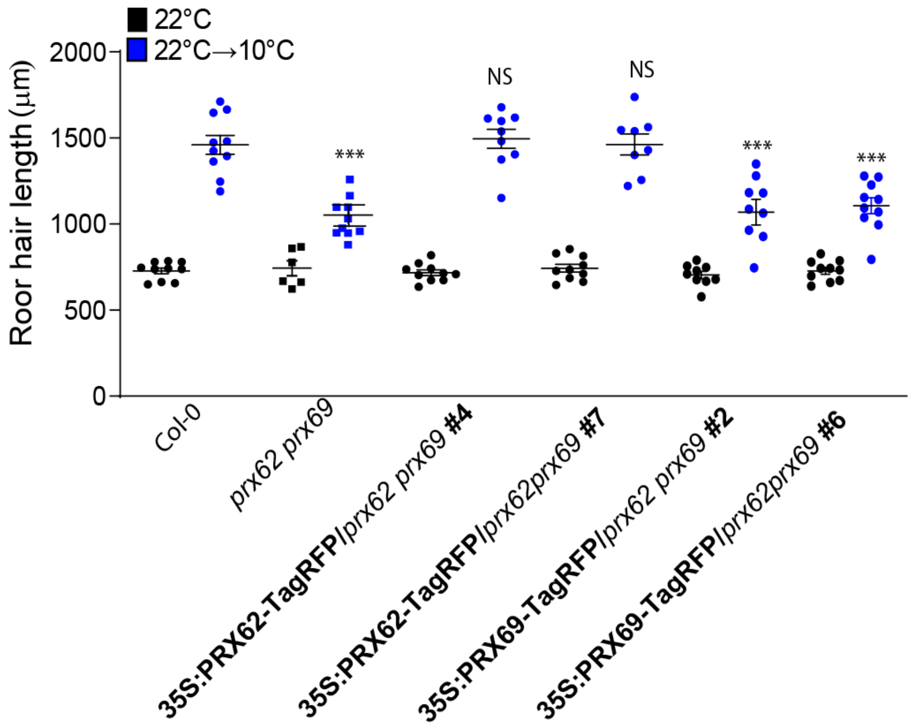

Supplementary Figure 9. Phenotypic rescue of *prx62-1 prx69-1* by overexpression of *PRX62* or *PRX69*.

35S:PRX62 is able to rescue RH growth of *prx62-1 prx69-1* double mutant while 35S:PRX69 fails to rescue RH growth of *prx62-1 prx69-1* double mutant. Each point is the mean of the length of the 10 longest RHs identified in a single root. Results are the mean of three biological replicates ± SD. Asterisks indicate significant differences between Col-0 and the corresponding genotype at the same temperature (two-way ANOVA followed by a Tukey–Kramer test; (***) $p < 0.001$). NS=non-significant differences. Exact *p-values* are provided in the Source Data file.

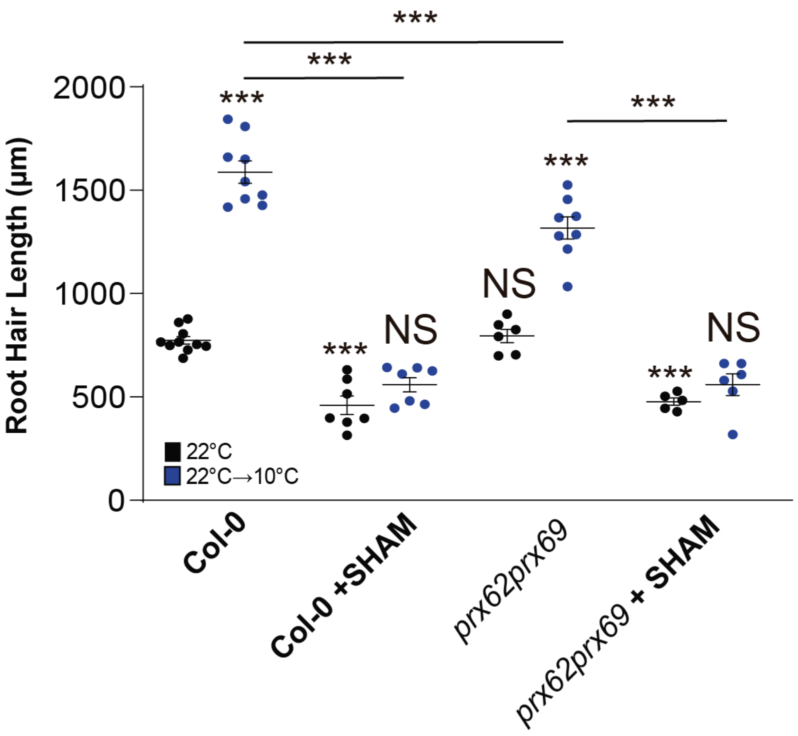

Supplementary Figure 10. Effect of SHAM on the double mutant *prx62-1 prx69-1*.

SHAM treatment (65 µM) on *prx62-1 prx69-1* double mutant have a strong effect on RH growth at low temperature. This suggest that other PRXs might be acting together with PRX62 and PRX69 on RH low temperature growth. Each point is the mean of the length of the 10 longest RHs identified in a single root. Data are the mean ± SD (N=15-20 roots), two-way ANOVA followed by a Tukey–Kramer test; (***) $p < 0.001$. Results are representative of three independent experiments. Asterisks indicate significant differences between Col-0 and the corresponding genotype at the same temperature. NS= non-significant differences. Exact *p-values* are provided in the Source Data file.

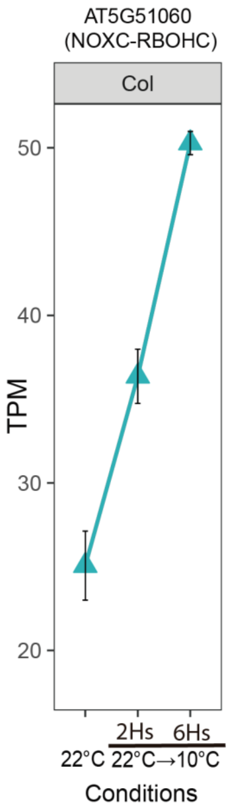

Supplementary Figure 11. Expression of *NOXC* (*RBOHC/RHD2*) under low-temperature assessed by RNA-seq. TPM = Transcripts Per Kilobase Million.

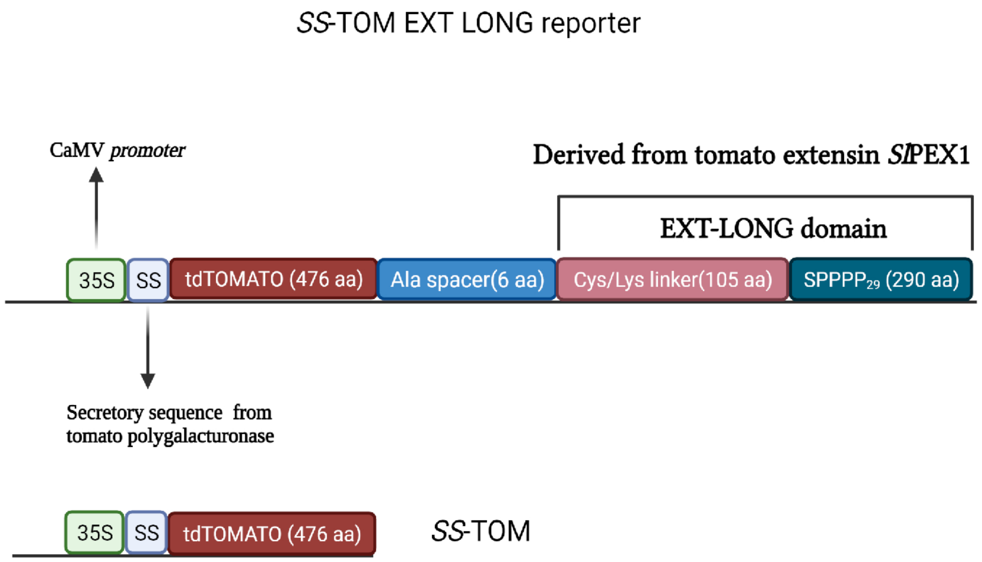

Supplementary Figure 12. **SS-TOM EXT-LONG and SS-TOM reporters**. Created with BioRender.com

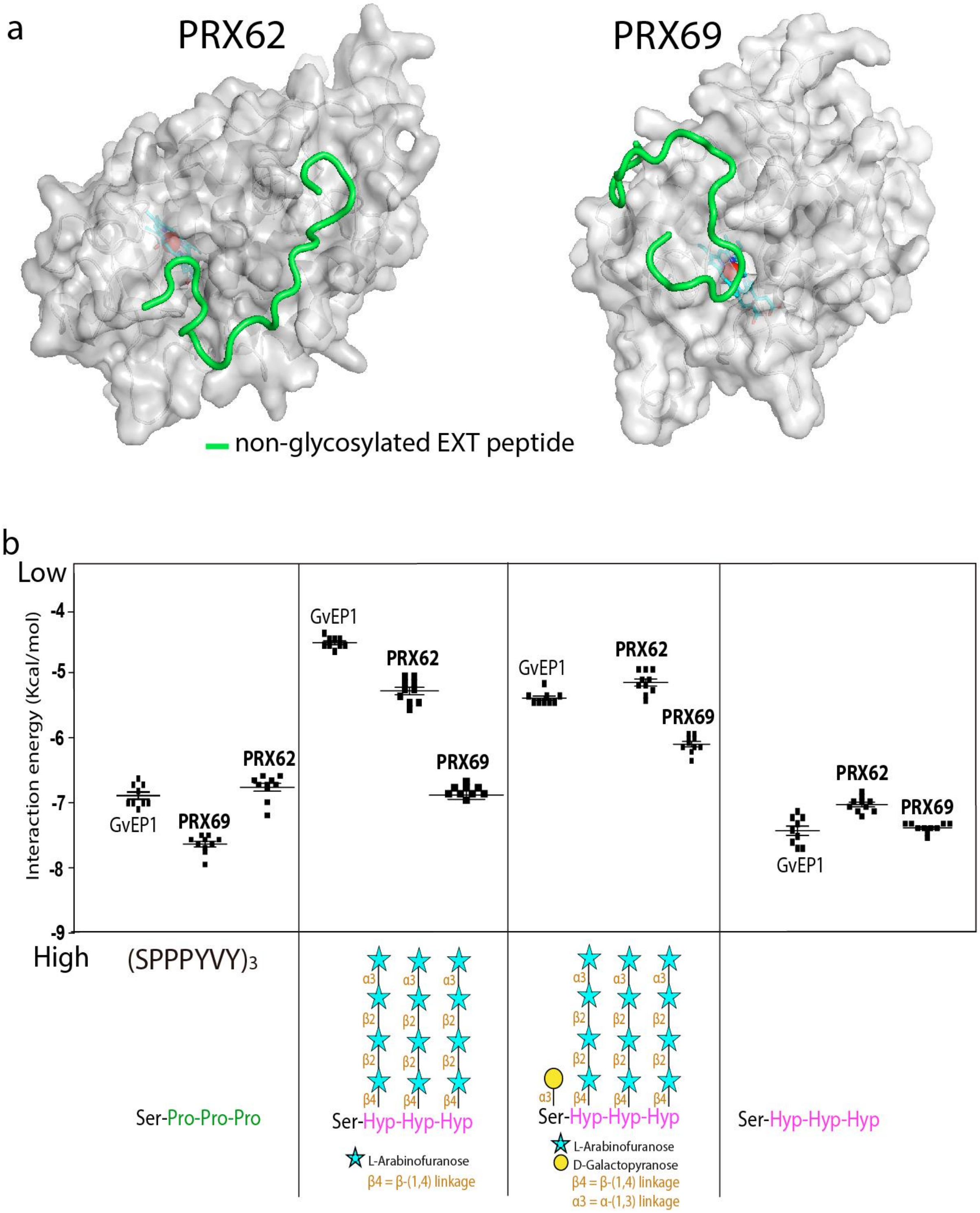

Supplementary Figure 13. Interaction by an *in silico* docking approach of PRX62 and PRX69 with EXT peptides.

(**a**) Models of PRX62 and PRX69 (protein surface shown in gray) based on their similarly to PRX24Gv from *Vitis vinifera* (peroxidase 24, GvEP1, LOC100254434). Homology modeling were performed for both PRXs using modeller 9.14 using the crystal structures 1PA2, 3HDL, 1QO4 and 1HCH as templates. PRX62 and PRX69 were complexed to a non-*O*-glycosylated EXT substrate $(SPPPYVY)_3$ (backbone depicted in green). Heme's solvent accesibilitiy can be seen in the middle of the pictures, as thin sticks with a central red sphere highlighting its iron as.
(**b**) Comparison of the binding energy (kcal/mol) of different peroxidases to EXT substrates with different degrees of *O*-glycosylation. A non-hydroxylated EXT peptide $(SPPPYVY)_3$, a hydroxylated but not *O*-glycosylated EXT peptide [$(SOOOYVY)_3$; O=hydroxyproline], only arabinosylated EXT-peptide [$(SOOOYVY)_3$-A], and arabino-galactosylated EXT peptide [$(SOOOYVY)_3$-AG] were analyzed.

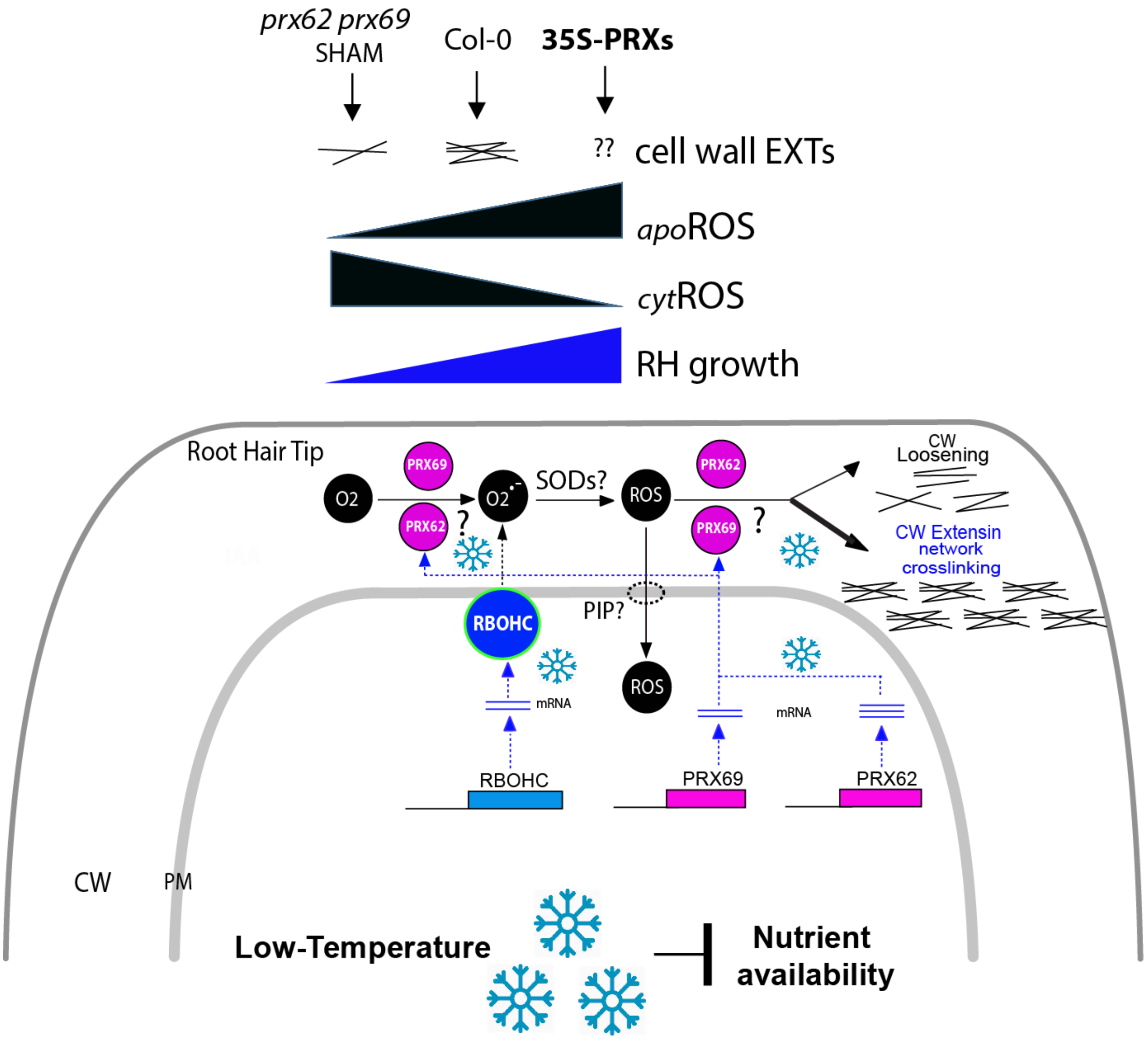

Supplementary Figure 14. **Proposed Model of PRX62 and PRX69 functions in ROS-homeostasis and EXT-cell wall association linked to RH growth at low-temperature**.

ROS-homeostasis ($_{apo}$ROS + $_{cyt}$ROS) in the RH tip. Higher $_{apo}$ROS/low $_{cyt}$ROS than Col-0 present in 35S$_{pro}$PRXs promotes RH growth while lower $_{apo}$ROS/high $_{cyt}$ROS *in prx62-1 prx69-1* represses RH growth at low-temperature. Changes in $_{apo}$ROS triggered by PRX62 and PRX69 might control changes in the EXT-mediated cell wall expansion/crosslinking in the apical RH zone. Part of the $_{apo}$ROS might be translocated to the cytoplasm to contribute to the $_{cyt}$ROS helped by Plasma membrane Intrinsic Proteins (PIPs) to be determined.

References

90. Fulton, L. M. & Cobbett, C. S. Two alpha-L-arabinofuranosidase genes in Arabidopsis thaliana are differentially expressed during vegetative growth and flower development. J. Exp. Bot. 54, 2467–2477 (2003).
