# Supplementary material for: Apoplastic class III peroxidases PRX62 and PRX69 promote Arabidopsis root hair growth at low temperature
Source: Nat Commun. 2022 Mar 14;13:1310. doi: 10.1038/s41467-022-28833-4 (PMC8921275; doi:10.1038/s41467-022-28833-4)
Supplement: Supplementary file 3 — Description of Additional Supplementary Files [file 41467_2022_28833_MOESM3_ESM.pdf]

Description of Additional Supplementary Files

File name: Supplementary Data 1

Description: Alignment of the of the forward DNA strand re-sequenced data from 852 accessions in PRX62 coding region and promoter region.
